# Implementing Technology Literacy Programs in Retirement Homes and Residential Care Facilities: Conceptual Framework

**DOI:** 10.2196/34997

**Published:** 2022-08-19

**Authors:** Karen S Li, Nathan Nagallo, Erica McDonald, Colin Whaley, Kelly Grindrod, Karla Boluk

**Affiliations:** 1 Department of Biology Faculty of Science University of Waterloo Waterloo, ON Canada; 2 enTECH Computer Club University of Waterloo Waterloo, ON Canada; 3 Department of Kinesiology and Health Sciences Faculty of Health University of Waterloo Waterloo, ON Canada; 4 School of Public Health Sciences Faculty of Health University of Waterloo Waterloo, ON Canada; 5 School of Pharmacy Faculty of Science University of Waterloo Waterloo, ON Canada; 6 Michael G. DeGroote School of Medicine McMaster University Hamilton, ON Canada; 7 Department of Recreation and Leisure Studies Faculty of Health University of Waterloo Waterloo, ON Canada

**Keywords:** older adult, technology, retirement home, long-term care, social connections, technology literacy program, retirement, elderly, literacy, implementation, concept, framework, knowledge translation

## Abstract

**Background:**

The COVID-19 pandemic caused widespread societal disruption, with governmental stay-at-home orders resulting in people connecting more via technology rather than in person. This shift had major impacts on older adult residents staying in retirement homes and residential care facilities, where they may lack the technology literacy needed to stay connected. The enTECH Computer Club from the University of Waterloo in Ontario, Canada created a knowledge translation toolkit to support organizations interested in starting technology literacy programs (TLPs) by providing guidance and practical tips.

**Objective:**

This paper aimed to present a framework for implementing TLPs in retirement homes and residential care facilities through expanding on the knowledge translation toolkit and the framework for person-centered care.

**Methods:**

Major concepts relating to the creation of a TLP in retirement homes and residential care facilities were extracted from the enTECH knowledge translation toolkit. The domains from the framework for person-centered care were modified to fit a TLP context. The concepts identified from the toolkit were sorted into the three framework categories: “structure,” “process,” and “outcome.” Information from the knowledge translation toolkit were extracted into the three categories and synthesized to form foundational principles and potential actions.

**Results:**

All 13 domains from the framework for person-centered care were redefined to shift the focus on TLP implementation, with 7 domains under “structure,” 4 domains under “process,” and 2 domains under “outcome.” Domains in the “structure” category focus on developing an organizational infrastructure to deliver a successful TLP; 10 foundational principles and 25 potential actions were identified for this category. Domains in the “process” category focus on outlining procedures taken by stakeholders involved to ensure a smooth transition from conceptualization into action; 12 foundational principles and 9 potential actions were identified for this category. Domains in the “outcome” category focus on evaluating the TLP to consider making any improvements to better serve the needs of older adults and staff; 6 foundational principles and 6 potential actions were identified for this category.

**Conclusions:**

Several domains and their foundational principles and potential actions from the TLP framework were found to be consistent with existing literatures that encourage taking active steps to increase technology literacy in older adults. Although there may be some limitations to the components of the framework with the current state of the pandemic, starting TLPs in the community can yield positive outcomes that will be beneficial to both older adult participants and the organization in the long term.

## Introduction

Older adults’ ability to stay socially connected has become more challenging as a result of the COVID-19 global pandemic. The World Health Organization and public health agencies in North America have advised all individuals to follow physical distancing measures and limit gatherings to minimize transmission of COVID-19 [1-3]. Older adults (individuals who are 65 years of age or older) are identified as a group at greater risk for severe illness from COVID-19, compared to the general public [4]. Throughout the course of the pandemic, long-term care (LTC) visitor restrictions have been implemented to protect the health of residents and staff members in these settings. Although it is important to limit the spread of COVID-19, these measures have disrupted the normal social routines of older adults, which may increase the risk of anxiety, depression, cognitive dysfunction, heart disease, and overall mortality [5,6]. Throughout the pandemic, the World Health Organization and public health agencies have encouraged individuals to maintain social connections through digital alternatives, using phones, computers, tablets, and other electronic devices, but little support is available to assist those who need help using technology for these purposes.

Previous research has identified that older adults are often interested in using technology for recreation, but feelings of apprehension and difficulty in accessing and using technology limit its uptake by older adults [7-9]. Existing literature suggests that active steps should be taken to educate and support older adults in their engagement with technology to help them build and maintain social connections with their family, friends, and the wider community [9-12]. Implementing teaching and education opportunities by volunteer organizations, for instance, that involve peer-to-peer learning and intergenerational relationships to help older adults adopt technology are recommended [5,9,13].

Although technology adoption in LTC settings increased during the pandemic, ensuring continued access to technology and technology education can maintain and increase its uptake by older adults [8]. In many regions, community organizations provide technology education and assistance to older adults at a low cost or for free, such as the enTECH Computer Club, based out of the University of Waterloo in Waterloo, Ontario, Canada. Before the pandemic, enTECH volunteers worked with residents of local LTC homes and supported older adults reaching their technology goals [13,14]. To facilitate the expansion of club activities to other locations, enTECH club members have also developed a knowledge translation toolkit in consultation with the University of Waterloo faculty [15]. The toolkit serves as a starting point for LTC homes to implement technology programming, consolidating much of the club’s work.

The purpose of this paper is to describe a framework to guide the implementation of technology programming in residential care facilities and retirement home settings, using the framework for person-centered care by Santana et al [16] as a template. To complement the toolkit by Nagallo et al [15], we aimed to develop a comprehensive framework with which a technology program can be started. Additionally, we wish to frame the development and rollout of a technology literacy program (TLP) as being fundamentally person focused, leading to the selection of the framework for person-centered care. Through consultation with the enTECH team, the TLP framework maps the knowledge translation toolkit onto the framework for person-centered care, in which it provides foundational principles and potential actions to guide the framework’s implementation. The implementation of technology literacy programming through this framework aims to encourage older adults to use technology and to create opportunities for fostering social connections and maintaining healthy aging.

## Methods

### Framework Model Exploration

**Figure 1:**
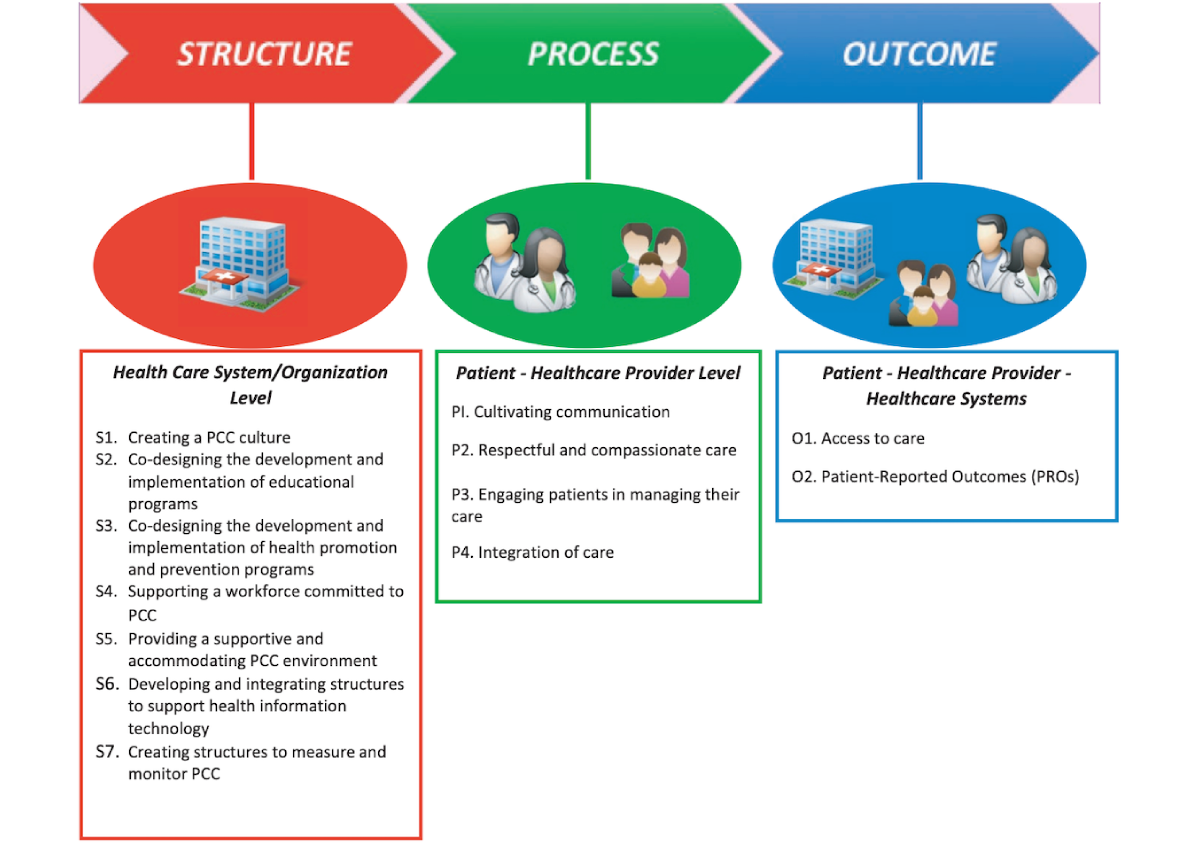
Framework for person-centered care (PCC) by Santana et al [16]. PRO: patient-reported outcomes.

A search using Google Scholar was conducted during June 2020 to search for existing frameworks involving or implementing change in health care contexts, particularly with the use of technology. The framework for person-centered care by Santana et al [16] (Figure 1) was identified as having general person-centered domains that could be consistent with a TLP program for older adults, the residential care facility, and its staff. The framework for person-centered care is itself based on the Donabedian Model [17] for the assessment of quality of care, which divides assessment into “structure,” “process,” and “outcome” components. The framework for person-centered care was selected over other frameworks, such as those applicable to adult education (eg, Kirkpatrick model [18]) or implementation (eg, RE-AIM [19,20]), due to a focus on implementation, person-centeredness, and provider-patient partnership [16]. The framework for person-centered care contains 13 domains split between 3 themes that serve as foundational pillars to implement person-centered care in a health care environment [16].

### Domain Mapping

**Figure 2:**
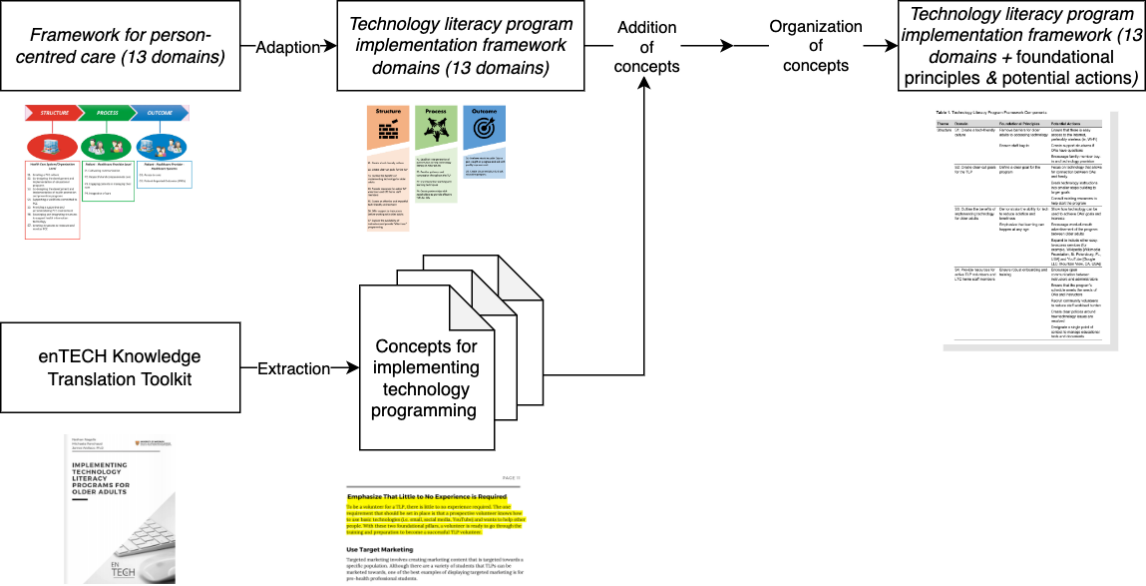
Process of developing the technology literacy program implementation framework.

The lead author of the knowledge translation toolkit (NN) modified the 13 domains from the framework for person-centered care to reflect the requirements of starting a TLP in a LTC setting. The language in framework for person-centered care by Santana et al [16] was adapted to focus on technology literacy programming, incorporating program implementation, technology education, quality assurance, and stakeholder relations [16]. To identify relevant foundational principles and potential actions for TLPs, the 13 domains for the TLP implementation framework were inserted into a Miro whiteboard (RealtimeBoard, Inc), a cloud-based collaborative whiteboard software. Foundational principles are defined as ideal steps to be taken to implement a successful TLP. Potential actions are defined as action items to help with a particular domain, but they are not completely mandatory for a TLP to be implemented. Author KL identified and extracted every major concept from enTECH’s knowledge translation toolkit, focusing on TLP; these concepts were organized under the 13 TLP domains. Authors NN, CW, and KL performed a final check of the domains and associated concepts on the Miro whiteboard. The resulting Miro whiteboard is shown in Multimedia Appendix 1. Authors KL, NN, EM, and CW finalized the categorization of the concepts into either foundational principles or potential actions for each domain. This process is detailed in Figure 2.

## Results

### Framework Overview

**Figure 3:**
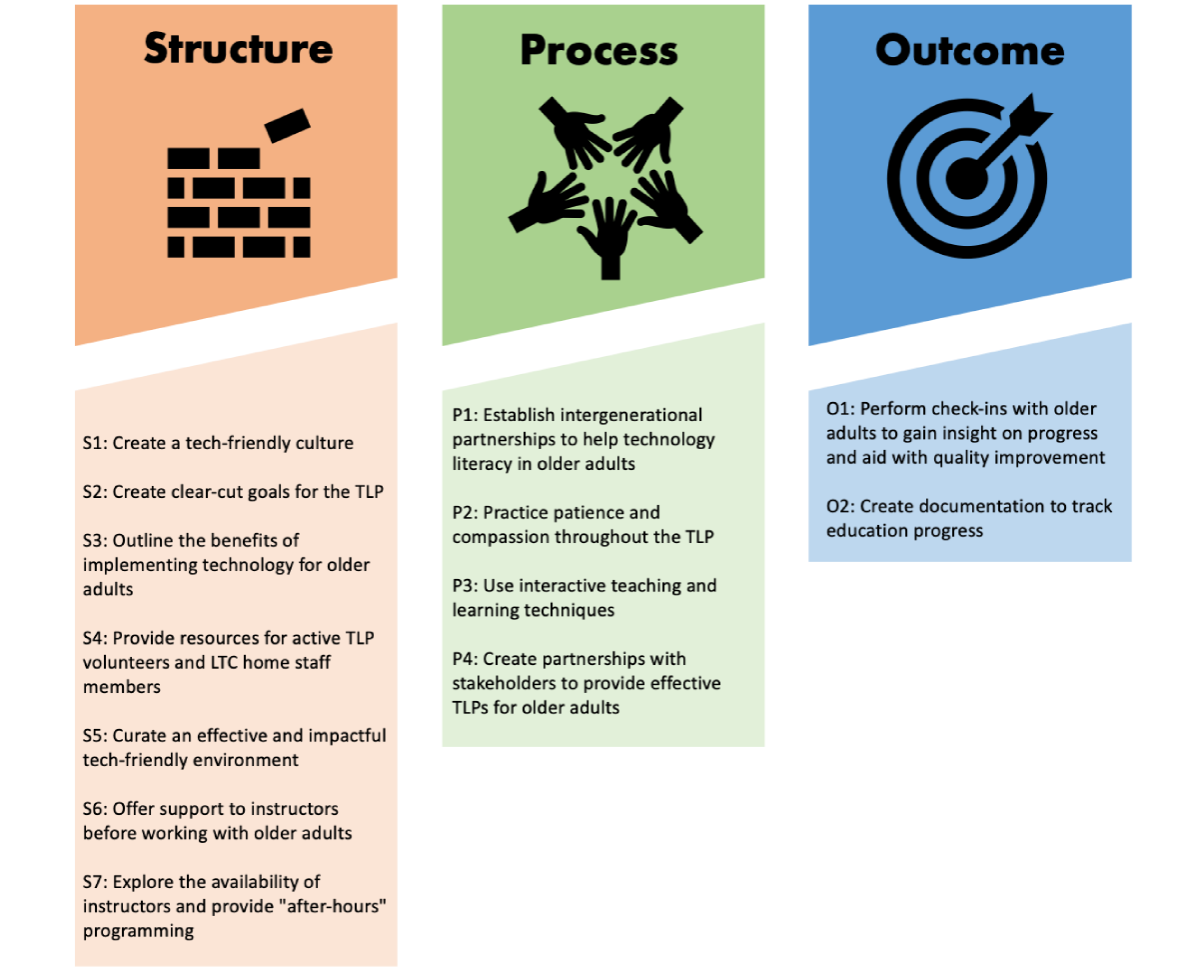
Technology literacy program (TLP) framework.

Similar to the framework for person-centered care [16], the TLP implementation framework uses the 3 main categories of “structure,” “process,” and “outcome” to sort the 13 domains needed to implement a TLP. Under these 3 categories, 7 domains were placed under “structure,” 4 domains were placed under “process,” and 2 domains were placed under “outcome.” To build this framework, domains were used to guide discussion about foundational principles and potential actions. The 3 main categories and their domains are organized in the order that they should be executed during the implementation process. The final TLP framework model is shown in Figure 3.

### Domains Overview

The TLP framework consists of 3 main categories that are essential to designate the domains and components of the framework; these 3 categories are “structure,” “process,” and “outcome.” The domains and components under each category are detailed in Tables 1-3.

### Structure (S1-S7)

According to Donabedian [17], “structure” defines the attributes of the setting in which care takes place. It describes the facilities, financials, human resources, and organizational structure of the place where care occurs. The framework for person-centered care also uses this definition to build their domains for this category [16]. As shown in Table 1, the “structure” category for TLP implementation focuses on developing organizational infrastructure to deliver a successful TLP. This category also includes strategies on how various resources may be used to serve as the foundation of TLP implementation.

**Table 1:** Structure domains and components of the technology literacy program (TLP) framework.

Structure domains	Foundational principles	Potential actions
S1: Create a tech-friendly culture	Remove barriers to accessing technology for older adults Ensure staff buy-in	Ensure that there is easy access to the internet, preferably wireless internet (ie, Wi-Fi) Create support structures if older adults have questions Encourage family member buy-in and technology provision
S2: Create clear-cut goals for the TLP	Define a clear goal for the program	Focus on technology that allows for connection between older adults and family Break technology instructions into smaller steps building to larger goals Consult existing resources to help start the program
S3: Outline the benefits of implementing technology for older adults	Demonstrate the ability for tech to reduce isolation and loneliness Emphasize that learning can happen at any age	Show how technology can be used to achieve older adults’ goals and interests Encourage word-of-mouth advertisement of the program between older adults Expand to include other easy-to-access services (eg, Wikipedia and YouTube)
S4: Provide resources for active TLP volunteers and long-term care homes staff members	Ensure robust onboarding and training	Encourage open communication between instructors and administrators Ensure that the program’s schedule meets the needs of older adults and instructors Recruit community volunteers to reduce staff workload burden Create clear policies around how technology issues are resolved Designate a single point of contact to manage educational tools and documents
S5: Curate an effective and impactful tech-friendly environment	Reduce paperwork burden on instructors, understanding that some documentation might be needed Ensure that technology is user-friendly and accounts for differences in strengths between individuals	Have resources that can be easily accessed by instructors, including printouts Advertise the benefits of technology to older adults via posters and town halls Consider a buddy or group system where older adults can be taught the same content together Listen to and act on what older adults themselves indicate they want to learn or achieve
S6: Offer support to instructors before working with older adults	Stress that the requirements to become an instructor are low: only basic technology literacy with common software (ie, email and YouTube) is needed	Ensure instructors have basic tech literacy skills to help older adults Create a straightforward onboarding process Host practice sessions with instructors (eg, on ethical scenarios and common technology problems) Use a web-based platform (eg, Slack and Microsoft Teams) where instructors can easily communicate, while maintaining confidentiality
S7: Explore the availability of instructors and provide “after-hours” programming	Schedule sessions at practical times and create opportunities for learning and follow-up if questions arise when there is no instructor available	Create a knowledge base that older adults can access if there are no instructors available or for self-study (eg, an easy-to-access website or binder) Reevaluate instructor availability frequently Ensure older adults can anonymously leave feedback about programming (eg, an anonymous comment box)

### Process (P1-P4)

The “process” category used in the framework for person-centered care includes the processes to deliver person-centered care [16]. Specifically, it describes strategies that can be used by health care practitioners to ensure person-centered care is being provided [16]. These steps closely resemble the action items to begin and maintain a TLP in LTC homes in the “process” category of TLP implementation. As depicted in Table 2, the domains in the “process” category outline procedures undertaken by stakeholders involved (eg, volunteers, club executives, resident facility staff, and older adults) to ensure a smooth transition from conceptualization into action.

**Table 2:** Process domains and components of the technology literacy program (TLP) framework.

Process domains	Foundational principles	Potential actions
P1: Establish intergenerational partnerships to help technology literacy in older adults	There is no set number of instructors needed to help set up a successful TLP Instruction can be in person, digital, or over video chat or phone	Encourage older adults to write any questions they have in between teaching sessions Network with other community groups to identify potential volunteers
P2: Practice patience and compassion throughout the TLP	Encourage older adults to protect their personal information, including financial information Encourage instructors to use their judgement and limit their support to their comfort level Ensure that instruction allows older adults to learn at their own pace Instructors can develop or improve their teaching, leadership, and communication skills	Repetition and practice are key to support learning and are sometimes overlooked
P3: Use interactive teaching and learning techniques	Programs can be one-on-one, in small groups, or lecture based, depending on older adults and instructor’s comfort and knowledge as well as room capacity limitations Facilitation must be sensitive to the needs of older adults, including vision, hearing, and mobility challenges Encourage instructors to adapt to different learning styles to assist older adults as best as possible Remember that layperson terms for instructors may not be layperson terms for older adults	Limit session to 1.5- to 2-hour blocks to minimize participant and instructor fatigue Have diverse learning resources available for older adults and instructors
P4: Create partnerships with stakeholders to provide effective TLPs for older adults	Identify what existing TLPs do in other settings to develop curriculum and foster participant engagement Be open to the potential for collaborating with existing organizations that provide digital services	Advertise this opportunity broadly; pre-health professional students may be particularly interested in participating in it Identify existing local supports for community programs, including organizations that may provide technology at a discounted price Reach out to long-term care or retirement home stakeholders and the community at large to see if any organizations are looking to liquidate older technology Designate one representative from your organization to liaise with other stakeholders for the purposes of acquiring technology donations and recruiting instructors

### Outcome (O1-O2)

In the framework for person-centered care, the “outcome” category describes the effects of person-centered care on patients [16]. It also describes performance indicators of person-centered care in hopes of measuring such indicators to identify areas for improvement to better serve patients [16]. Table 3 reflects how TLP implementation shares similar attributes with the “outcome” category, as it focuses on how TLPs can benefit older adults even with diminished volunteer capacity, and how quality improvement can be used to adjust programming to better serve the needs of the community.

**Table 3:** Outcome domains and components of the technology literacy program framework.

Outcome domains	Foundational principles	Potential actions
O1: Perform check-ins with older adults to gain insight on progress and aid with quality improvement	Older adults’ progress on accomplishing goals can be tracked using a web-based spreadsheet like Google Sheets Focus on the person not the technology; technology should always come second Validated tools like the Single Ease Question [21] can be used to evaluate the difficulty of a task Questions or questionnaires to be filled out by older adults can be hosted via the internet, using Google Forms, to encourage older adults to practice their tech skills	Check in with older adults to see how they feel about their progress A brief informal postsession feedback question is recommended, even a simple “did you like this session today?”
O2: Create documentation to track education progress	Consider curriculum and training documents as “living documents,” and iterate on them based on feedback A “best practices” guide can be used to structure lessons more generally	Lesson plans can be used to teach content and can optionally be used by residents in the absence of instructors Provide training documents to instructors Platforms like Google Docs and Notion (Notion Labs Inc) can be used to organize documents and can be updated immediately Tracking students’ progress and interests can facilitate transition between different instructors

## Discussion

### Overview

This paper outlines an implementation framework for TLPs in LTC and residential homes and with older adults. Building on a person-centered care framework, the TLP framework starts by outlining how the program should be structured to create a safe learning environment for older adults to learn to use technology. The framework also outlines processes that can be used to build confidence and competence among both older adult learners and instructors, including multigenerational pairings, focusing on web-based safety, and ample hands-on learning opportunities. The framework concludes with 2 domains under “outcome” that are focused on identifying the benefits to older adults and opportunities for quality improvement.

### Structure

Domains S1 to S7 are aimed to create a safe learning environment for older adults to learn how to use technology. Having a positive initial experience supported by a well-established, friendly, and supportive learning environment can promote continual participation of the TLP and continual use of technology among older adults, and so domain S1 reinforces this [22]. Domain S3 concentrates on informing older adults of the benefits of implementing technology in their lives. Beyond increasing technology literacy, existing research has suggested that learning new skills and keeping an active mind may maintain cognition and mental health throughout life [23]. In addition, the “structure” category of the framework aims to reduce the social isolation and loneliness that older adults may experience during the pandemic through delivering interactive learning [24,25]. Many of the foundational principles and potential actions of domain S7 were consistent with existing findings, including those suggesting a strong training preference for self-teaching through methods such as reading a printed manual and learning through “trial and error” or “playing around” approach [22,26]. Although self-practicing after a lesson can be beneficial, the TLP framework focuses on the value of instructors to teach older adults and facilitate their learning in TLPs (domains S7 and O1). There is a focus on providing resources for program leaders so that they can effectively support older adults, as emphasized in domains S4 through S7.

### Process

Domains P1 to P4 outline the means by which TLP should be operated. This includes engaging older adult learners with staff of TLP within retirement homes and residential care facilities to lead to a person-centered programming. A study conducted during the COVID-19 pandemic suggested that LTC facilities should have dedicated staff to assist residents in using information communication technologies that are already provided by the facilities, such as tablet devices [27]. Therefore, domain P1 has considered this by creating a group of instructors to teach older adult learners and facilitate the TLP operations. Existing cognitive aging research has outlined recommendations on techniques that instructors may find useful when teaching older adults to use computer software applications [28]. Many of the “process” domains align with recommendations based on that research [28]. Additional research identified reasons for older adults’ negative attitudes about technology use, which were frequently associated with inconvenience with the technology device, disliking the features of technology, and security and reliability concerns [29]. Given these concerns, foundational principles from domain P2 emphasize the importance of protecting personal and financial information. Social contact or other social interactions have been shown to be the second most important motivator for older adult learners to participate in learning activities [30]. Therefore, domains P2 and P3 aim to have older adults develop healthy interpersonal relationships with instructors to foster participant engagement. Another study that examined teaching modality themes used by student mentors to help older adults learn technology reinforces the foundational principles and the overall theme of domains P2 and P3 [22]. Finally, other research has suggested social engagements may also maintain cognitive aging and lower mortality outcomes as much as physical activity, depending on the level of social activity [31].

### Outcome

Domains O1 and O2 aim to evaluate the progress of the TLP and determine if it is meeting older adults’ goals to learn technology. Existing research has recognized the need for technological training programs focused on older adults to evaluate and analyze the effectiveness of teaching technology to them [32]. To enhance the experience of TLP for staff and older adults, TLPs must determine the positive feedback and improvements that can be made. The insights received from following the foundational principles and potential actions of domain O2 allow for more research to be conducted to determine the strengths and improvements needed for TLP.

### Strengths and Applicability of the Framework

Governments and health care systems have been encouraging people to remain engaged with their communities via the internet during the COVID-19 pandemic [1-3]. The implementation of TLPs for older adults may help them overcome some of the known barriers to engage with technology. Increased technology literacy, fostered through TLP, may also support older adults in accessing health care digitally, an adoption of which has been accelerating in response to the pandemic [33,34].

It has been suggested by findings of a recent study that using a person-centered care approach to engage older adults with technology is crucial in creating meaningful technology-mediated enrichment experiences for them [35]. The TLP framework supports this approach as it aims to implement fundamentally person-focused TLPs, hence the framework for person-centered care was chosen as the template [16]. By organizing the domains’ content into foundational principles and potential actions, we hope that organizations can identify parts of the framework that are readily applicable and can be implemented with little difficulty, which can be beneficial during times of high visitor restrictions. For instance, during visitor restrictions in LTC, organizations may begin with creating a knowledge base that older adults can access with minimal assistance if there are no instructors available, which is a potential action from domain S7. In addition, organizations can plan opportunities to expand their existing TLPs. Lastly, there is a focus on creating and externalizing partnerships to run the program to reduce costs and foster new relationships with others in the community.

### Limitations

Despite the benefits of implementing TLPs using this framework, there are some drawbacks to consider. The COVID-19 pandemic has highlighted how complex it can be to support older adults through periods of intense isolation. Periods of visitor and volunteer service restrictions, such as local or global disease outbreaks, can present challenges to the implementation of TLPs. For instance, older adults may have to ask LTC staff outside of TLP for help, and this may interfere with the techniques they learned with their usual TLP instructors. Organizations implementing TLPs could consider incorporating a group of on-site staff who can assist older adults during periods of visitor and volunteer service restrictions if they see fit. Although these events cannot easily be anticipated aside from typical seasonal patterns (ie, increased probability of influenza outbreaks in fall and winter months), ensuring TLP participants are sufficiently trained such that they can accomplish basic communication tasks independently can foster independence and reduce feelings of isolation.

Furthermore, the authors’ experience of TLPs are based on implementing TLPs directly and assisting with situating them in existing retirement and LTC settings as an external organization. The authors do not have experience implementing a TLP as an employee of a retirement home or LTC home. EnTECH’s experience in TLP implementation is also limited to Ontario, Canada. Thus, the generalizability of this TLP framework may be limited, as the structure of residential care facilities and retirement homes outside of Ontario may differ from those in Ontario. Finally, despite the value of technology, the financial cost of the devices and additional equipment to run a TLP is a concern in both private and publicly funded residential care facilities, and that is addressed in domain P4.

### Future Research

Further research should be conducted to validate the utility of this framework in retirement homes and residential care facilities. This could include evaluating the success in implementing a TLP in retirement homes and residential care facilities according to the insights on the progress and quality of the program gained from the check-ins with older adults (domain O2). Additionally, there is an interest in exploring whether older adults are able to independently apply the technology literacy skills they have developed or improved from their TLP to achieve and expand on their technology use. After the implementation of a TLP in retirement homes and residential care facilities, the publication of case studies aimed at both industry and academics is suggested to raise awareness of this framework for residential care employees and stakeholders.

### Conclusions

Recognition in staying socially connected through technology has undoubtedly increased throughout the course of the COVID-19 pandemic. A framework for implementing TLPs was developed to support retirement homes and residential care facilities that are interested in starting their own TLP to support older adults’ technology competence. The foundation of the framework was constructed from enTECH’s knowledge translation toolkit for implementing TLPs for older adults, and it was structurally organized by following the framework for person-centered care by Santana et al [16]. Although modifications to the framework may be required depending on an organization’s needs, starting TLPs for older adults can have potential positive impacts on them in the long term.

## Appendix

Multimedia Appendix 1 Domains and their foundational principles of the technology literacy program framework on Miro whiteboard.

## Abbreviations

LTC: long-term care
TLP: technology literacy program
