# Supplementary material for: Implementing Technology Literacy Programs in Retirement Homes and Residential Care Facilities: Conceptual Framework
Source: JMIR Aging. 2022 Aug 19;5(3):e34997. doi: 10.2196/34997 (PMC9440411; doi:10.2196/34997)
Supplement: Multimedia Appendix 1 [file aging_v5i3e34997_app1.pdf]

Target: LTC homes who want to start technology literacy programs (TLPs) for their residents during the COVID-19 pandemic

Sticky Note Legend
White = from Nate's KT project
Yellow = Execs
Purple = Nate + Karen

13 domains = goals
stickies = actionable elements

- Note: instructors = staff/volunteers

Instructions:

- Take the pre-written stickies based on Nathan's KT project and arrange them into the new domains
- Make your own sticky notes and arrange them as well
- As a group we will come to a concensus re: all sticky placements

Original framework by Santana et al:

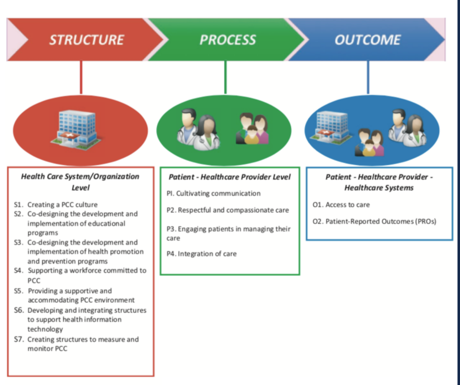

FIGURE 1 Framework for person-centred care

Activities from Nate's KT Project:

Make your own! Please only do so after taking a look at the existing activities!

← Click on the sticky, and click cmd/ctrl-D or right-click and dulplicate!

Structure

S1': Create a tech-friendly culture

- a way to remove barriers for older adults to use technology
- Having easy access to tech
- Access to WiFi communally
- ensuring staff-buy-in: no one should talk badly about it!
- Knowing that people are there to support you if you have questions
- encourage family members to bring in tech or get on board with setting up something for the older adult

S2': Create clear-cut goals for TLP

- Help older adults bridge the gap between reducing social isolation and the use of technology through implementing your own Technology Literacy Program (TLP)
- focus on technology that focuses on keeping elder residents connected with loved ones
- Breaking a big task into smaller steps
- Align goals of the older adult with the program
- Define some clear goal - this will look different for different contexts!
- enTECH's KT can be used as a reference when creating these goals

S3': Outline the benefits of implementing technology for older adults

- Show how tech can be used to achieve older adults' goals and interests
- Connect feelings of isolation or loneliness to ways technology can help
- word of mouth from other older adults
- Expand beyond only video call/message - Wikipedia and YouTube are easy for most folks to understand!
- it's always good to learn a new skill! ; emphasize that there is no age limits to learning and using technology

S4': Provide resources for active TLP volunteers and LTC home staff members

- Open communication between staff involved in program rollout and admin is important
- Timing of programs needs to reflect older adults' schedules and volunteers' availability
- Volunteer-managed program is good, as staff have other duties
- Determine what onboarding of the program looks like for established staff members
- A way for probelms to be actioned: OA, tech and infrastructure-wise
- clear educational tools includes documents, videos,and all accessible in one platform

S5': Currate an effective and impactful tech-friendly environment

- Admin burden should be on staff as little as possible, understanding that record keeping may be important (ie, list of emails and pass)
- Have resources that are easily available (i.e. printouts with instructions, unlocked computers) so individuals can quickly get help/ don't have to ask for help
- Advertise how technology can be utilized, create programs that utilize technology (buddy system, programs that demonstrate the value of tech)
- Listen to what the older adult wants to learn/achieve
- technology and software is user-friendly and accounts for OAs needs, disabilities, etc.

S6': Offer support to volunteers before working with older adults

I am wondering how this could happen within the COVID context (we may need to adapt this point)

volunteers and/or staff members working w/ tech

- prospective volunteer knows how to use basic technologies and wants to help other people
- Create on-boarding process as straightforward as possible
- there is little to no experience required to become a volunteer for a TLP
- Emphasize that the software used is basic: YT, wikipedia, skype, etc
- Do practice with volunteers (ethical scenarios)
- create a platform where volunteers and staff can easily interact (ex. Slack) or trade contact info while maintaining confidentiality

S7': Create documentation (ex. educational/knowledge tools, quality improvement (QI) to track education process

- lesson plans used to teach curriculum and to be used for residents without volunteer supervision
- Training docs are provided to volunteers
- iterate documents based on OA interest and staff feedback
- Google Docs and Notion are great free tools to organize info which can be updated immediately
- can create a "best practices" guide to help older adults with tech

Process

P1': Establish inter-generational partnerships to help technology literacy in older adults

- no set number of volunteers to help set up a successful TLP
- Connecting with local Universities to inquire ab volunteer interests.
- This doesn't have to include in-person visits: skype/phone options are totally valid!
- Reach out to individuals and organizations via doing outreach in the community (ex. doing presentations at retirement homes, universities, etc.)

P2': Practice patience and compassion through TLP

- encourage them to protect their financial information
- use your judgement and only help out with what you are comfortable with
- encourage own pace for older adult learning technology.
- Repitition and practice are key, and often overlooked
- volunteers can develop or improve on their teaching, leadership, and communication skills

P3': Use interactive teaching & learning techniques

- 2-hour time blocks have been used by us
- Programs can be 1:1, small group or lectures, depending on infection control, older adult knowledge and staffing
- sensitive to needs of older adult (hearing deficits, vision difficulties, etc.) and accomodating for them in your teaching
- diversity in learning resources
- Volunteers can adopt to different learning styles to teach and assist OAs as best as possible
- layman's terms for you may not be layman's terms for OA's - keep that in mind!
- communication is important! (especially when helping OAs remotely)

P4': Create partnerships with stakeholders to provide effective TLPs for older adults

- The best targeted marketing is pre-health professional students
- See what existing programs do in other contexts
- Utilize tutorials and virtual supports that are freely available (maybe highlight that many volunteer groups are pivoting to offer virtual supports)
- open promotion to all students of all programs
- See if there are donations that can be solicited from local businesses for older tech
- point of contact was an LTC employee in charge of recreational programming volunteer coordinating
- 1 liaison from the LTC should act as point of contact to TLP and speak on behalf of rest of TLP execs.

S1 and S6 are similar

perhaps merging two would be a better option since they are both similar domains

Outcome

O1': Explore availability of active volunteers and innovate "after-hour" learning

- develop a knowledge base of common issues are questions from lived experiences
- Reaching out to old volunteers about their availability every few months/term
- create interactive study guides that OAs can access when not speaking to someone directly
- Place for questions to be asked if the program is not runnning (ie, box, form to fill out)
- Have a space where older adults can provide feedback or concerns about the tech environment and receive non-judgemental support/ anonymously leave points for improvement
- Recieve prompt questions to questions asked "after-hours"

O2': Perform check-in's with older adults to gain insight on progress and aid with QI

- Progress and important information is tracked on an online spreadsheet
- Check in with Older adults to see how they feel on thier own progress.
- Remember that the technology is not the most important piece of the puzzle: building confidence/providin g motivation etc.
- Do some sort of evaluation of the program, even if a simple "did you like this sesion" today?
- Can use "single ease question" (or a form of it):

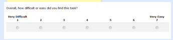

- allow them to practice their tech skills by accessing and completing a feedback form
